# Bio-efficacy of aluminum phosphide and cypermethrin against some physiological and biochemical aspects of *Chrysomya megacephala* maggots

**DOI:** 10.1038/s41598-023-31349-6

**Published:** 2023-03-16

**Authors:** Mahran Tony, Mahmoud Ashry, Mohammad M. A. Tanani, Abdelbaset M. A. Abdelreheem, Mohammad R. K. Abdel-Samad

**Affiliations:** 1 Department of Zoology, Faculty of Science, Al-Azhar University, Assiut, Egypt; 2 Department of Zoology and Entomology, Faculty of Science, Al-Azhar University, Nasr City, Cairo, Egypt

**Keywords:** Zoology, Entomology

## Abstract

Carrion flies play a significant role in forensic entomotoxicology, where they are employed as alternative samples when traditional samples are unavailable. In situations of poisoned death, these toxins disrupt insect development and affect forensic entomology analyses. So, forensic entomotoxicologists must be aware of this impact. The present study aimed to determine the effects of aluminum phosphide (AlP) and cypermethrin (CP) on the biochemical parameters and antioxidant enzymes of the third instar of *Chrysomya megacephala* maggots. *C. megacephala* was reared on normal and poisoned rabbit carcasses with aluminum phosphide and cypermethrin. The third larval instar of *C. megacephala* was studied using by spectrophotometer for detection of total protein, (TP), aspartate aminotransferase (AST), alanine aminotransferase (ALT), total antioxidant capacity (TAC), superoxide dismutase (SOD), glutathione s-transferase (GST), catalase (CAT) and malondialdehyde (MDA). The results indicated to significantly decrease of TP, TAC, SOD, GST and CAT and increase of AST, ALT and MDA in the maggots reared on the poisoned carcasses with AlP or CP compared with control group. In conclusion, the tested insecticides brought about a decrease antioxidant enzyme activity and increase of MDA could be involved in free radicals in *C. megacephala* larvae leading to oxidative stress by these insecticidal components.

## Introduction

The oriental latrine fly, *Chrysomya megacephala*, a member of the family Calliphoridae, is a necrophagous dipteran, one of the dominant flies of forensics, thus used in forensic investigation^1^. This species is widely distributed over the world, and causing accidental myiasis^2^. The potential use of blowfly maggots as indicators for detecting toxins and drugs in decomposing carcasses have been widely demonstrated^3^.

The insect life cycle stage that feeds on the cadaver is a potential reservoir of undigested flesh from the corpse, because, in some circumstances, the flesh from the corpse can retain some types of toxins that had been consumed by the victim before death and which may even have been the cause of death, these toxins may be recovered by analyzing the insects^4^. Several researchers^5,6^ detected several toxins, drugs, and pesticides in different species of fly larvae that fed on intoxicated corpses.

Pesticides are chemical substances that are used to eradicate pests such as insects, rodents, fungus, and unwelcome plants (weeds). Pyrethroid pesticides are widely used in insect control owing to their low toxicity in mammals and relatively low environmental impact^7,8^. Pyrethroid are effective against a broad spectrum of insects, even in low doses and the lethal action on insects involves peripheral and central neurons^9,10^. Certain examples of pyrethroids used for insect control are cypermethrin, permethrin, and deltamethrin^11^. Despite they beneficial effects, they unmanaged and repeated applications result in unexpected consequences in non-target organisms^12^. Cypermethrin for example, a class II pyrethroid pesticides, has toxic effects on nervous system^13^ as well as on the functions of other systems and organs including such as liver^14,15^.

Aluminum Phosphide (AlP) is another pesticide that is used for protecting crops during repository and hipping. Accidental and suicidal human toxicity with AlP in most cases ends with death^16^. In insects, pesticides cause oxidative stress and lipid peroxidation^17^. Peroxidation and enzymatic activity levels are crucial in estimating toxin stress^18^.

There have been insufficient previous studies on the maggots of *C. megacephala* that fed on intoxicated carcasses. The aim of this study was to evaluate the effects of aluminum phosphide and cypermethrin on some metabolic parameters and antioxidant enzymes of the last instar maggots of *Chrysomya megacephala*.

## Results

### Total protein quantification (TP)

**Figure 1:**
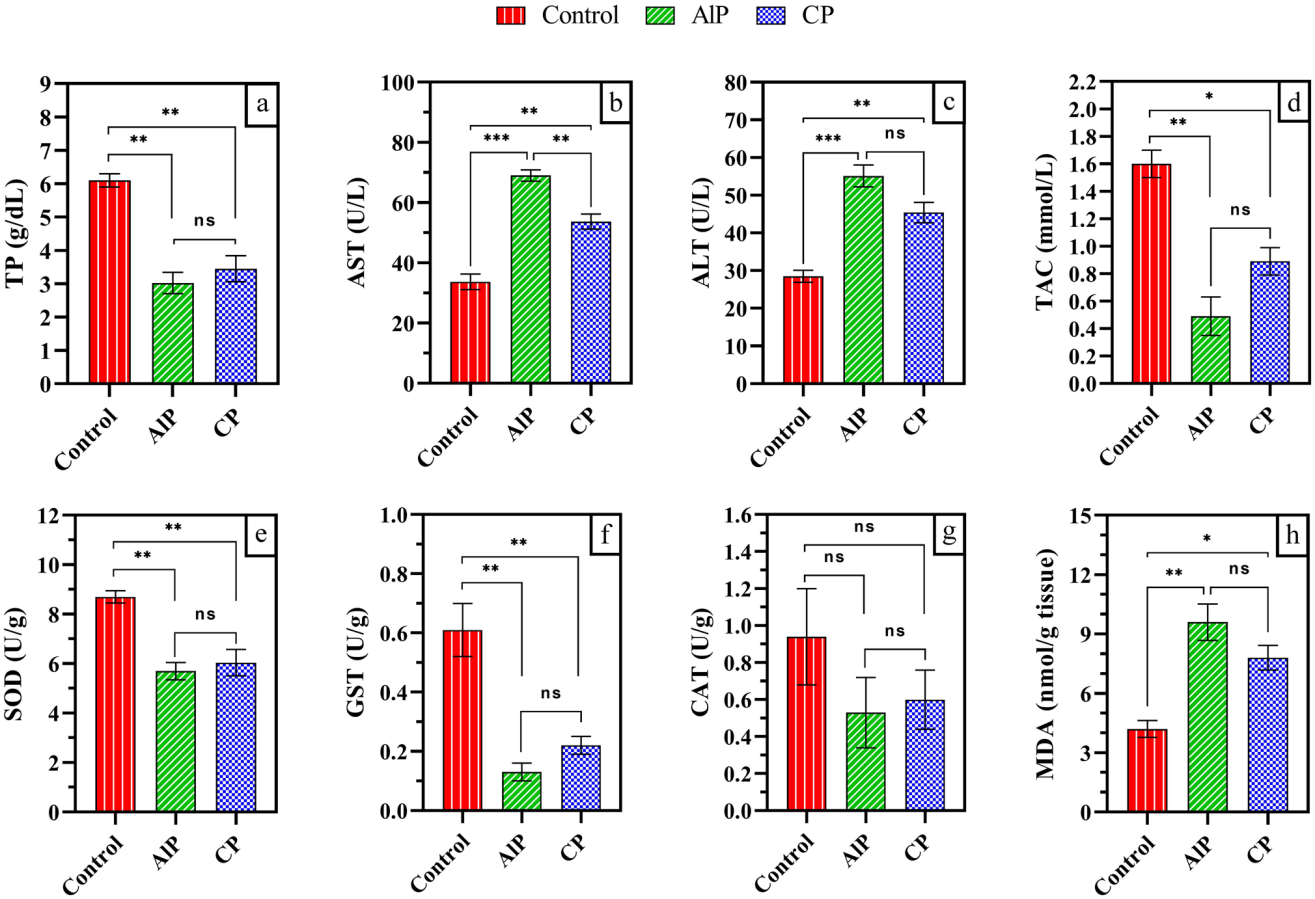
Impact of aluminum phosphide (AlP) and cypermethrin (CP) pesticides on the biochemicals parameters of *Chrysomya megacephala*. (**a**) total protein (TP); (**b**) aspartate aminotransferase (AST); (**c**) alanine aminotransferase (ALT); (**d**) total Antioxidant Capacity; (**e**) superoxide dismutase (SOD); (**f**) glutathione S- transferase (GST); (**g**) catalase (CAT); (**h**) total lipid peroxidation (MDA). *p < 0.05; **p < 0.01; ***p < 0.001; *ns* not significant.

Protein synthesis is necessary for the maintenance of body growth and reproduction. Many factors had been implicated in the control of protein synthesis. According to the data assorted in (Fig. 1a). AlP and CP caused remarkable decrease in the total protein level of larvae forced to eat on treated carcasses compared to the total protein in larvae of the control carcasses. Recorded total protein was 3.02 ± 0.32, 3.45 ± 0.39 and 6.1 ± 0.20 g/dL in the larvae of AlP, CP and control groups, respectively.

### Aspartate aminotransferase (AST)

As shown in Fig. 1b. Significant increase in the AST level was detected in the maggots reared on AlP carcasses compared to the maggots on the control carcasses. The AST for the AlP and control groups was 69.01.9 and 33.72.6 U/L, respectively. In the same context, CP caused a steady increase in AST level. Where the AST level in the maggots reared on CP carcasses reaching 53.72.5 U/L.

### Alanine aminotransferase (ALT)

As clearly shown in Fig. 1c, maggots fed on AlP and CP carcasses had a significant increase in ALT levels when compared to maggots grown on control carcasses. The maggots in AlP and CP and control groups recorded ALTs of 55.1 ± 2.9, 45.4 ± 2.7 and 28.5 ± 1.6 U/L, respectively.

### Total antioxidant capacity (TAC)

The obtained data showed significant decrease in the TAC level for the maggots fed on AlP carcasses (TAC: 0.49 ± 0.14 mmol/L) and CP carcasses (TAC: 0.89 ± 0.10 mmol/L) compared with the TAC level for the maggots fed on control carcasses (TAC: 1.60 ± 0.10 mmol/L) as illustrated in Fig. 1d.

### Superoxide dismutase (SOD)

Superoxide dismutase (SOD) levels in the maggots fed on AlP and CP carcasses were recorded 5.7 ± 0.35 and 6.04 ± 0.53 U/g**,** respectively. These levels were significantly lower than the SOD level for maggots fed on control carcasses (8.70 ± 0.25 U/g), as shown in Fig. 1e.

### Glutathione S- transferase activity (GST)

As illustrated in Fig. 1f, the obtained data showed that the maggots fed on AlP and CP carcasses showed a significant decrease in the glutathione S- transferase (GST) level that was 0.13 ± 0.03 and 0.22 ± 0.03 U/g, respectively. This decrease is significance decrease with compared with the GST level (0.61 ± 0.09 U/g) for maggots in the control group.

### Catalase (CAT)

The results showed no significance differences between the catalase (CAT) levels of maggots in all groups (Fig. 1g). CAT level was 0.94 ± 0.26, 0.53 ± 0.19 and 0.60 ± 0.16 (U/g) for maggots fed on control, AlP and CP carcasses, respectively.

### Total lipid peroxidation (Malondialhyde; MDA)

The disturbance of lipid content of maggots fed AlP and CP carcasses was varied according to the potency of insecticides as well as the developmental age. As presented in Fig. 1h, The maggots that fed on control, AlP and CP carcasses showed 4.20 ± 0.40, 9.60 ± 0.92 and 7.80 ± 0.62, respectively in the level of MDA. Significant increase was recorded in the level of MDA in the maggots that fed on AlP and CP compared to MDA level in the maggots in control group. At the same time, no significance difference detected between the MDA levels in maggots of AlP and CP groups.

## Discussion

Pesticides come in over a thousand distinct varieties and are applied all over the world. Pesticides are used in agriculture to kill pests that harm crops and in public health to kill disease-carrying vectors like mosquitoes^19^. In agriculture sector, Pesticides are widely used and are an effective and cost-effective strategy to improve agricultural output quality and quantity^20^. Pesticides such as pyrethroid were used on a variety of vegetable crops^21^. They may also be used to combat cockroaches^22^, mosquitoes^23^, fleas, and termites^24^ in households as well as other buildings^25^.

Another low-cost, effective and widely used pesticide is aluminum phosphide (AlP). However, among agricultural pesticides, it is currently one of the most prevalent causes of poisoning^26^. Several incidents of persistent poisoning or mortality has been documented and proved among agricultural workers exposed to various types of pesticides in developing nations. These incidents mostly resulted from use/misuse of pesticides^27^.

In cases of poisoned death, these poisons disrupt insect development (such as Carrion flies) and have an impact on forensic entomology investigations (especially when traditional samples of victim are unavailable). In light of this, forensic entomotoxicologists need to be aware of this effect.

Generally, a significant biochemical effect was induced on maggots after fed on treated carcasses by AlP and CP. In the current investigation, the TP content decreased significantly in the maggots of treated groups compared to control group. While the activity of AST and ALT was increased in the treated maggots compared to control. These changes have been supported by previous studies that showed decrease in TP and increase in ALT and AST activities^28–30^. Proteins are biological macromolecules made up of one or more long chains of amino acids. Protein synthesis is required for growth and reproduction. Proteins are required for cell division and to govern chemical reactions throughout the cell metabolic process^31^.

Protein content may be attributed to the disenchantment of protein biosynthesis by amino acids and the reduction in protein could be attributed to changes in protein and free amino acid metabolism and their synthesis^32,33^. In addition, the observed decrease in proteins could be attributed in part to the damaging effect of insecticides as cypermethrin on larvae tissue cells, as confirmed by the increase in activities of AST, ALT for treated groups than control^34^. Also, Kinnear et al.^35^ suggested that decreased protein levels were due to decreased synthesis of new proteins by the fat body, haemolymph and other tissues of the *Calliphora stygia* larvae.

Similar literatures are in harmony with these results. Total protein levels in *Culex fatigans* were decreased by λ-cyhalothrin^36^. The level of total proteins was decreased in *Bacterocera zonata* pupae resulted of larvae treated with Beticol, Biosad, Elsan, Lufox, Mani, Match and Radiant during all tested periods as compared to control^37^. Total protein content in *Culex pipiens* declined while total lipids increased gradually with proceeded the generations^38^.

Detoxification enzymes in insects are important tools in defense mechanisms against toxins and xenobiotics. They play valuable roles in upholding the normal physiological status^39^. Detoxifying enzymes have been found to respond against insecticides, or compounds exhibiting insecticidal activities. The detoxifying enzymes were found to counter against insecticides activities^29^. Induction of detoxification metabolic system plays an important role in insect's detoxification mechanism^40^. In addition, decreasing ACP activity might be due to the reduced phosphorus liberation for energy metabolism and decreased rate of metabolism, as well as decreased rate of transport of metabolites^41^.

In our results, observed an increase in lipid peroxidation (MDA) and also in, AlP and CP exposure to the larvae diminished the activities of enzymatic antioxidants, superoxide dismutase (SOD), TAC, catalase (CAT) and glutathione S transferase (GST), increasing of Oxidative enzyme malondialdhide (MDA) that indicates to lipid peroxidation. Enzymatic and non-enzymatic systems preserve the antioxidant status, these defense systems become decreased during oxidative stress, leading a metabolic derangement due to an imbalance caused by excessive generation and diminished antioxidant capacity followed by larvae cell injury and inflammation^42,43^**.** Also, the antioxidant enzymes such as SOD, CAT and GST play a vital role in relief and prevent tissue toxicity and inflammation^44,45^, a higher effect observed in larvae treated with AlP, where the latter enzymes showed a moderate decrease in their levels regarding CP group may be attributed to its inhibition effect on free radical formation or through scavenging the well-formed radicals and it was deficiency might be associated with development of treated larvae. These findings are consistent with Gheldof et al.^46^, who stated that antioxidant analysis of the different honey fractions revealed that the water soluble fraction contained the majority of the antioxidant components, including gluconic acid, ascorbic acid, hydroxymethylfuraldehyde, and the enzymes glucose oxidase, catalase, and peroxidase.

Our results in contrast with other study that showed that increase of antioxidant enzyme activity during the harmful effect of dichlorvos, the SOD enzyme plays a critical role in ROS defence^47^. Previous research found that activation of SOD activity is the primary response of lepidopteran insects to organophosphate poisoning and other dietary prooxidants^48,49^.

In our study, CAT activity was decreased as compare to control, the decrease of CAT activity, may be due to the fact that CAT is known to be inhibited by superoxide anion buildup during degradation activities, which may be caused by insecticide and increased production of free radicals may lead to depletion or inactivation of CAT enzyme, and this due to high level of superoxide radical generation during oxidative stress in the acute stage of *C. megacephala*^42,47,50^.

GST activity in *C. megacepala* larva after treatment showed a significant decrease changes in treated groups compared with control these results were in agreement with Linares et al.^44^ and Doğru-Abbasoğlu et al.^51^, due to is involved in the inactivation of toxic lipid peroxidation products accumulated during destructive processes caused by this insecticides, according to the above results, AlP and CP increased free radicals (MDA) and decreased antioxidant defense in the larval haemolymph, hence, the viability of larvae decreased, the developed yield in larvae stages rearing decreased also. On the other hand, GST plays an important role on the biotransformation of both xenobiotic and endogenous substances. Therefore, its inhibition and induction has been used as a biochemical marker of exposure to xenobiotics with electrophilic centers^52,53^.

The pattern of the motor signs after AlP and CP administration is strongly suggestive of CNS toxicity^54^. Activities of SOD, CAT, GSH, and MDA levels in the larvae reflect the oxidative status and the haemolymph enzymes like AST and ALT, represent the functional status of the larvae^55^. Chemical-induced cellular alteration varies from simple increase of metabolism to death of cell and the increase or decrease of enzyme activity is related to the intensity of cellular damage^56^.

Therefore, increase of transaminase activity along with the decrease of activity of free radical scavengers may be the consequence of AlP and CP induced pathological changes of the larvae and the decreased CAT and SOD activities and increased MDA level in the larvae as well as increased AST and ALT levels suggest that insecticides causes damage in larvae tissues which may be through free radicals, in oxidative stress producing depletion of the activity of CAT, SOD, and the glycogen level, and increased level of MDA leading to tissues necrosis^57^. And in acute toxicity study, single dose of AlP and CP in increased the levels of MDA, AST and ALT and decreased the activities of TAC, SOD, GST and CAT level in treated larvae.

In conclusion, the both AlP and CP can affect third-instar maggots of *C. megacephala*. Antioxidant enzyme marker such as, TAC, SOD, GST and CAT and oxidative stress of (MDA) were involved in the free radical to these toxin exposure in maggots of *C. megacephala* and disturbances in the development of insect survived of treatments and they might be involved in integrated pest management strategies.

## Materials and methods

### Pesticides

Aluminum phosphide tablets (Shijiazhuang Awiner Biotech Co., Ltd) and cypermethrin (cypermethrin CP, > 99% pure, Gharda Chemicals Ltd. Mumbai) were used.

### Flies’ origin and laboratory colony

Rotten chicken viscera organs were employed as bait to catch adult *Chrysomya megacephala* flies^58^. *C. megacephala* was taxonomically classified using taxonomic keys^59^. According to earlier researches^60,61^, the flies were raised in a controlled laboratory conditions with mild adjustments. Breifly, the flies were housed in hardwood cages (40 × 40 × 40 cm^3^) that were provided with the necessary meal at a temperature of 25 ± 2 °C, relative humidity of 60 ± 10%, and a light–dark cycle of 12:12 h.

### Animal model

This experiment was carried out on nine mature male domestic rabbits weighing approximately 1.5 kg. The rabbits were housed in adequate cast steel cages for 15 days for acclimation at 25 ± 2 °C, 12:12 h light–dark cycle, and 60 ± 10% relative humidity. Across the experiment, all rabbits were kept in equivalent conditions of temperature, light, noise, and ventilation, and they were fed the same meal. Rabbit housing, procedures, and care principles were all carried out in accordance with the laboratory animal care and use recommendations^62^. The authors followed the ARRIVE standards.

### Experimental technique

Post acclimatization, the rabbits were randomly divided into three equal groups. 1st group represent the control group, in this group the rabbits were given distilled water. In 2nd group, the rabbits were treated with AlP (32.8 mg AlP/kg rabbit body weight)^63,64^. While the rabbits of 3rd group were treated with CP (1.4 mL/kg rabbit body weight)^65^. Carcasses of rabbits were moved into plastic boxes (25 × 10 × 15 cm^3^) that placed in the cages of flies to allowing the flies colonizing of the carcasses.

### Preparation of samples for analysis

The 3rd larval instars from each treated and control groups were collected then washed with saline, dried. About 100 mg from each collected larvae were homogenized in 1 mL of phosphate buffer. The samples were then centrifugated for 15 min at 14,000 r.p.m at 4 °C. The supernatant was stored at − 80 °C until analysis.

### Assessment of metabolic aspects

Total protein was evaluated by the method of Mæhre et al.^66^ and Zheng et al.^67^. Aspartate transaminase (AST) and alanine transaminase (ALT) were determined as described by Reitman and Frankel^68^, while glutathione-S-transferase enzyme (GST) activity was measured by methods of Pabst et al.^69^. Estimation of catalase (CAT) was done by method of Lück^70^.

Moreover, superoxide dismutase (SOD) was measured by methods of Misra and Fridovich^43^. Total antioxidant capacity and malondialdehyde (MDA) were estimated according to the methods of Koracevic et al.^71^ and Placer et al.^72^, respectively.

### Statistical analysis

The data were reported as the mean ± standard error of the mean (S.E.M). GraphPad Prism version 8.0.0 (for Windows, GraphPad Software, San Diego, California, USA) was utilized to conduct statistical analysis using one-way Analysis of Variance (ANOVA). p < 0.05 was considered significant.

### Ethical approval

Accepted in ethical committee at Faculty of Science (Assuit), Al-Azhar University. Certificate reference number: AZHAR10/2022.

## Data Availability

The datasets generated during and/or analyzed during the current study are available from the corresponding author on reasonable request.
